# Supplementary material for: Optimized 3D-Printed Polylactic Acid/Graphene Oxide Scaffolds for Enhanced Bone Regeneration
Source: Bioengineering (Basel). 2025 Nov 1;12(11):1192. doi: 10.3390/bioengineering12111192 (PMC12649579; doi:10.3390/bioengineering12111192)
Supplement: Supplementary file 1 [file bioengineering-12-01192-s001.zip › Supplementary Table S1.pdf]

930 μm

	1	2	3	4	5	6	Mean	SD
Yield Displacement (mm)	0.95	0.46	0.91	0.97	0.79	0.46	0.76	0.22
Yield Load (N)	556.42	422.29	650.83	545.08	471.71	422.29	511.44	81.71
Yield Strain (%)	3.29	1.40	3.11	3.41	2.65	1.41	2.54	0.84
Yield Strength (MPa)	2.96	2.17	3.46	3.02	2.58	2.20	2.73	0.46
Elastic Modulus (MPa)	157.22	163.02	173.24	149.74	135.68	165.79	157.45	12.13

690 μm

	1	2	3	4	5	6	Mean	SD
Yield Displacement (mm)	0.79	0.70	0.75	0.85	0.93	0.93	0.83	0.09
Yield Load (N)	1101.26	588.50	670.69	617.05	835.50	998.22	801.87	194.17
Yield Strain (%)	2.43	2.94	3.20	3.35	2.94	2.55	2.90	0.33
Yield Strength (MPa)	5.36	4.10	4.68	4.03	4.11	4.09	4.39	0.48
Elastic Modulus (MPa)	278.41	172.78	176.61	181.40	205.62	225.60	206.74	36.96

562 μm

	1	2	3	4	5	6	Mean	SD
Yield Displacement (mm)	0.67	0.77	0.99	1.10	0.74	0.85	0.86	0.15
Yield Load (N)	393.50	464.84	433.76	521.16	479.41	336.59	438.21	60.05
Yield Strain (%)	2.36	2.77	3.74	4.34	1.60	2.96	2.96	0.89
Yield Strength (MPa)	2.20	2.64	2.66	3.31	1.16	1.76	2.29	0.69
Elastic Modulus (MPa)	166.77	165.81	124.90	109.14	154.09	110.07	138.46	24.64

558 μm

	1	2	3	4	5	6	Mean	SD
Yield Displacement (mm)	0.88	1.10	0.86	0.77	0.91	1.04	0.93	0.11
Yield Load (N)	496.43	549.47	233.97	513.14	489.05	543.26	470.89	108.25
Yield Strain (%)	3.09	4.08	2.80	1.74	3.25	3.73	3.11	0.74
Yield Strength (MPa)	2.84	3.27	1.09	1.52	2.78	3.09	2.43	0.82
Elastic Modulus (MPa)	150.97	128.40	75.21	160.48	142.89	160.64	136.43	29.52

Supplementary Table S1. Compression test results of four types of scaffold material. Six specimens per pore size. Values are presented as mean ± standard deviation (SD).
