# Supplementary figures and images for: Optimized 3D-Printed Polylactic Acid/Graphene Oxide Scaffolds for Enhanced Bone Regeneration
Source: Bioengineering (Basel). 2025 Nov 1;12(11):1192. doi: 10.3390/bioengineering12111192 (PMC12649579; doi:10.3390/bioengineering12111192)

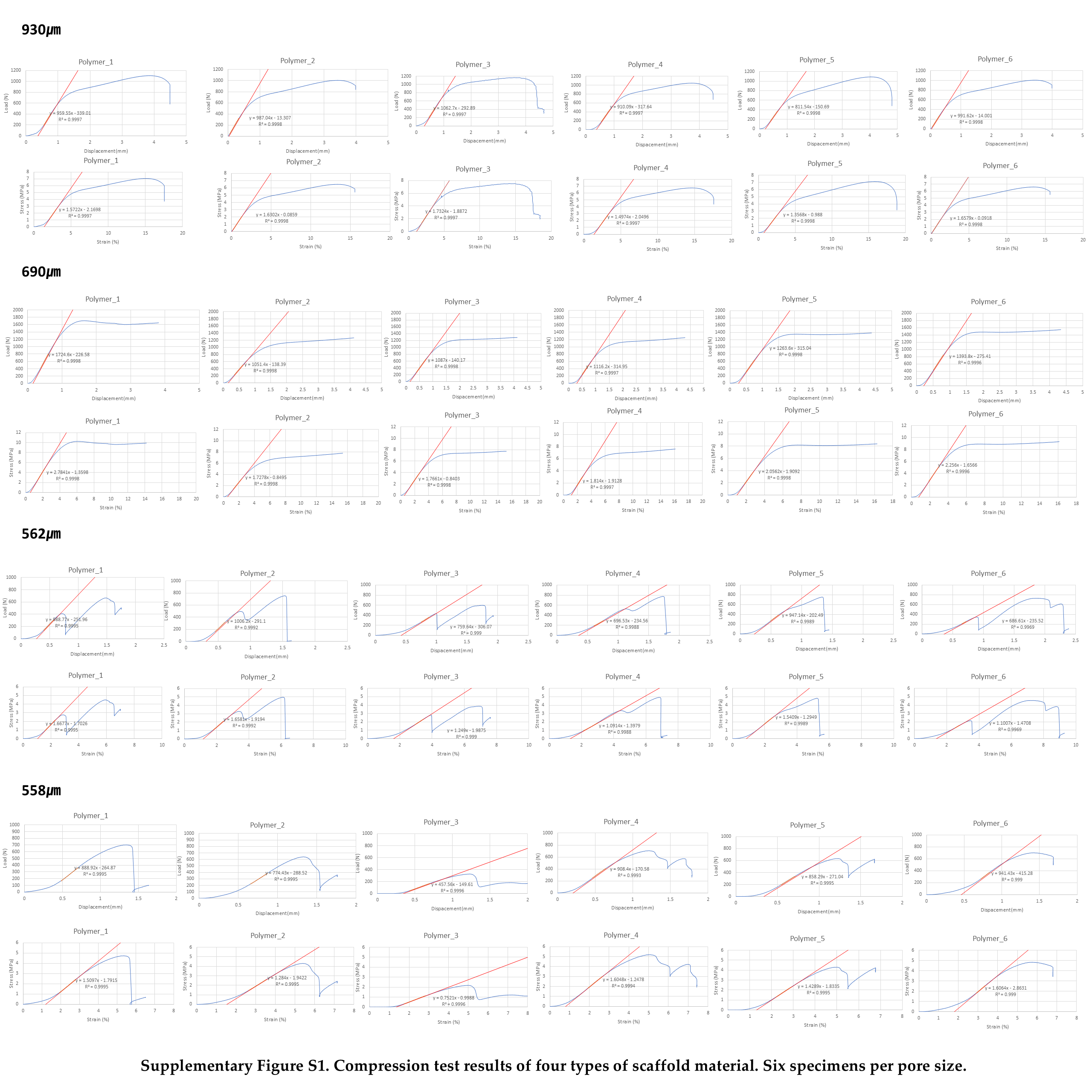

Supplement: Supplementary file 1 [file bioengineering-12-01192-s001.zip › Supplementary Figure S1.tif]
